# Diagnostic accuracy of DNA methylation in detection of gastric cancer: a meta-analysis

**DOI:** 10.18632/oncotarget.22613

**Published:** 2017-11-03

**Authors:** Weiling Hu, Wenfang Zheng, Qifang Liu, Hua Chu, Shujie Chen, John J. Kim, Jiaguo Wu, Jianmin Si

**Affiliations:** ^1^ Department of Gastroenterology, Sir Run Run Shaw Hospital, Medical School, Zhejiang University, Hangzhou, China; ^2^ Institute of Gastroenterology, Zhejiang University, Hangzhou, China; ^3^ Division of Gastroenterology, Loma Linda University Medical Center, Loma Linda, CA, USA

**Keywords:** gastric cancer, DNA methylation, blood, non-invasive, diagnosis

## Abstract

Emerging studies demonstrate the diagnostic utility of DNA methylation-based blood test for gastric cancer. The aim of the meta-analysis is to evaluate the accuracy of blood DNA methylation markers for detecting patients with gastric cancer. A systematic literature search to November 2016 that evaluated DNA methylation markers utilizing blood specimen to detect gastric cancer were selected to derive pooled sensitivities and specificities. 32 studies including 4,172 patients (gastric cancer (*N* = 2,098), control (*N* = 2,074)) met the study criteria. Overall sensitivity of DNA methylation-based blood test for detecting gastric cancer was 57% (95% CI 50–63%); specificity was 97% (95% CI 95–98%). Among patients who received plasma-based testing, sensitivity was 71% (95% CI 59–81%); specificity was 89% (95% CI 78–94%). Among patients who received serum-based testing, sensitivity was 50% (95% CI 43–58%); specificity was 98% (95% CI 96–99%). Using multiple methylated genes had sensitivity of 76% (95% CI 64–84%); specificity of 85% (95% CI 65–95%). DNA methylation test had sensitivity of 55% (95% CI 47–64%) and specificity of 96% (95% CI 92–98%) for detecting TNM stage I+II gastric cancer. In conclusion, blood-based DNA methylation test had high specificity but modest sensitivity for detecting gastric cancer. Evaluating multiple methylated genes or using plasma sample may improve the diagnostic sensitivity.

## INTRODUCTION

Gastric cancer is a common cancer worldwide and is associated with high morbidity and mortality. Approximately 984,000 incident cases of gastric cancer and 841,000 attributable deaths were estimated globally in 2013, which ranks fifth in cancer incidence and second in cancer-related deaths [1]. Although the incidence of gastric cancer has decreased in recent years, lack or non-specific symptoms among patients with early stage cancer prevents early detection and treatment in the majority of the patients [2]. Although accurate for detecting early gastric cancer, the cost and invasiveness of endoscopy as a primary screening test has been limited in large-scale screening programs [3].

In order to improve the detection of early gastric cancer, non-invasive blood biomarkers have received great interest. Although biomarkers such as carcinoembryonic antigen (CEA), carbohydrate antigen-19-9 (CA 19-9), and carbohydrate antigen-72-4 (CA 72-4) have been evaluated for diagnosis and surveillance of gastric cancer, the low sensitivity of these biomarkers do not warrant their routine use in clinical settings [4–6]. Emerging studies have highlighted that carcinogenesis of gastric cancer involves multiple processes that includes epigenetic as well as genetic alterations. Specifically, DNA methylation can lead to inactivation or activation of cancer-related gene [7, 8]. Furthermore, methylation of the promoter region can silence tumor suppressor genes that play an important role in regulating DNA repair, cell adhesion, cell-cycle regulation, signal transduction, and apoptosis [9, 10]. Modification in DNA methylation (e.g. Reprimo, hMLH1) frequently detected in gastric cancer tissues as well as in serum or plasma specimens in patients with gastric cancer, while seldom or absent in controls, suggests its potential application as a non-invasive biomarker [11, 12]. More importantly, several studies have demonstrated the presence of gene methylation in intraepithelial neoplasia further supporting the role of DNA methylation as an early event in the carcinogenesis of gastric cancer [13, 14].

Previous studies evaluating DNA methylation to differentiate patients with or without gastric cancer are limited by small sample size or inconsistent results. Therefore, we performed a meta-analysis to assess the diagnostic accuracy of DNA methylation markers as a non-invasive biomarker in detecting gastric cancer.

## RESULTS

### Literature search

**Figure 1:**
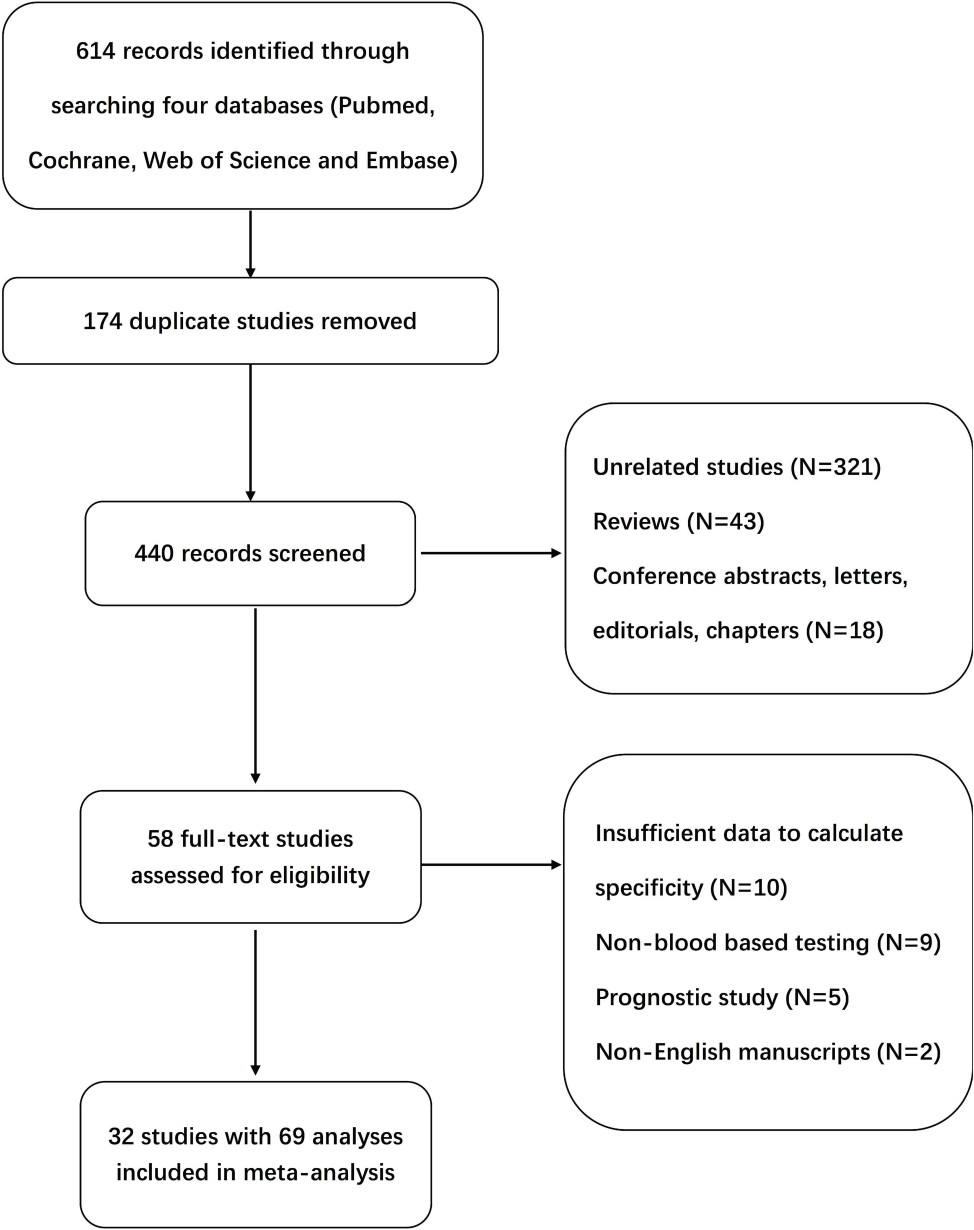
Flow diagram of studies identified in the meta-analysis

After a thorough literature search, we identified 197 records from Pubmed, two from Cochrane, 204 from Web of Science, and 211 from Embase. Of the 614 studies, 556 were excluded after reviewing the title and abstract for the following reasons: duplicate studies in 174, unrelated to study aim in 321, review papers in 43, meeting abstracts in 14, editorials in two, letter to the editor in one, and a book chapter in one. When 14 meeting abstracts were reviewed in detail, two were published in full-text and the data had been included in the analysis. However, the remaining 12 meeting abstracts were confirmed to not meet the study criteria (unrelated study in six, insufficient data to calculate study outcome in four, insufficient data to assess bias in two). Additional 26 studies were excluded after reviewing the full text (insufficient data for calculating specificity in 10, non-blood based testing in nine, prognostic study of patients with gastric cancer in five, and non-English manuscript in two). Finally, 32 studies that include 69 analyses of blood DNA methylation tests for evaluation of gastric cancer were included in the meta-analysis. The flow chart of the search method is shown (Figure 1).

### Characteristics of selected studies

The characteristics of the 32 publications including 69 analyses that compared frequency of DNA methylation markers between gastric cancer and control patients are shown in Table 1. Of the 32 included studies, nineteen evaluated methylation status of one gene, five evaluated two genes, and eight evaluated three or more genes. A total of 39 genes methylation were analyzed in these studies. P16 was reported in seven studies. E-cadherin and RASSF1A were reported in four studies. Finally, DAPK, APC, hMLH1, SFRP2, RUNX3, Reprimo, and RNF180 were reported in two studies.

**Table 1:** Characteristic of included studies

Author	Year	Country	Sample	Genes	Method	Case (N)	Control (N)
Lee TL, et al.	2002	China	Serum	DAPK/E-Caderin/p16/p15	MSP	54	30
Kanyama Y, et al.	2003	Japan	Serum	P16	MSP	60	16
Ichikawa D, et al.	2004	Japan	Serum	P16/E-Cadherin	MSP	109	10
Koike H, et al.	2004	Japan	Serum	p16/E-cadherin/ RARbeta	MSP	41	10
Leung YY, et al.	2005	China	Serum	APC/E-cadherin/ hMLH1/ TIMP3	MSP	60	22
LIU YH, et al.	2005	China	Plasma	P16	MSP	84	15
Cheng YY, et al.	2007	China	Serum	SFRP2	MSP	18	18
Tan SH, et al.	2007	Singapore	Serum	RUNX3/P16/RASSF1A/ CDH1	MSP	4	10
Bernal C, et al.	2008	Chile	Plasma	Reprimo	MSP	43	31
Abbaszadegan MR, et al.	2008	Iran	Serum	P16	MSP	52	50
Wang YC, et al.	2008	China	Serum	RASSF1A	MSP	47	60
Chen Z, et al.	2009	China	Serum	Hsulf-1	MSP	20	21
Guo X, et al.	2010	China	Plasma	IRX1	MSP	15	10
Zheng Y, et al.	2011	China	Serum	BX141696/WT1/CYP26B1/KCNA4	MSP	46	76
Ng EKO, et al.	2011	China	Plasma	SLC19A3	MSRED-qPCR	20	20
Chen L, et al.	2012	China	Serum	FAM5C/MYLK	MSP	58	30
Rajkumar T, et al.	2012	India	plasma	ATP4B	MSP	25	9
Cheung KF, et al.	2012	China	Plasma	RNF180	q-MSP	32	64
Ling ZQ, et al.	2013	China	Serum	XAF1	rt-MSP	202	88
Lu X, et al.	2012	China	Serum	RUNX3	MSP	202	852
Lee HS, et al.	2013	South Korea	Plasma	mSEPT9	MSP	153	96
Balgkouranidou I, et al.	2013	Greece	Serum	SOX17	MSP	73	20
Zhang H, et al.	2014	China	Blood	SPG20	MSP	41	21
Zhang X, et al.	2014	China	Plasma	RNF180/DAPK1/SFRP2	MSP	57	42
Balgkouranidou I, et al.	2015	Greece	Serum	APC/RASSF1A	MSP	73	20
Chen X, et al.	2015	China	Plasma	Zic1	MSP	104	20
Liu C, et al.	2015	China	Serum	SFRP1	MSP	42	20
Wang G, et al.	2015	China	Serum	FLNC/THBS1/UCHL1/DLEC1	q-MSP	82	86
Liu L, et al.	2015	China	Plasma	Reprimo/hMLH1	MSP	50	30
Xue WJ, et al.	2016	China	Serum	RASSF10	BSP	82	50
Pimson C, et al.	2016	Thailand	Plasma	PCDH10 /RASSF1A	MSP	101	202
Li WH, et al.	2016	China	Serum	OSR2/VAV3/ PPFIA3	MSP	48	25

These studies were conducted in nine countries (China, Japan, Thailand, Chile, Iran, Greece, South Korea, India, Singapore) and were published between 2002 and 2016. Of the 32 studies, 13 studies reported age and 15 studies reported gender data only among patients with gastric cancer while none of the studies provided age and gender data on control patients. Methylation-specific polymerase chain reaction was used in 31 studies to detect DNA methylation in the serum or plasma samples. In addition, 27 studies qualitatively analyzed the frequency of methylation.

### Diagnostic accuracy of DNA methylation markers in gastric cancer

**Figure 2:**
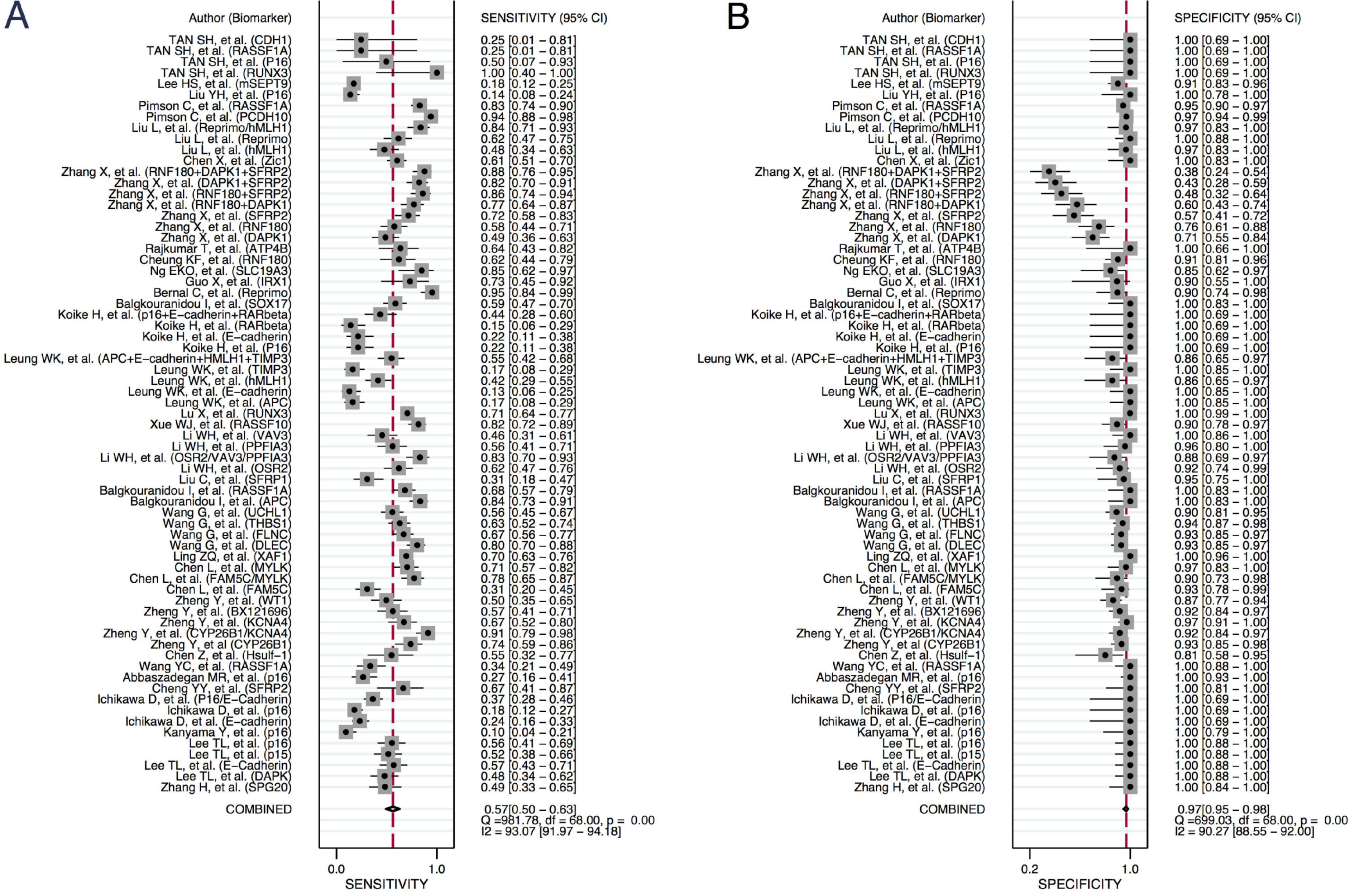
Forest plot of individual study and pooled sensitivities (**A**) and specificities (**B**) of blood DNA methylation marker for detection of gastric cancer.

**Figure 3:**
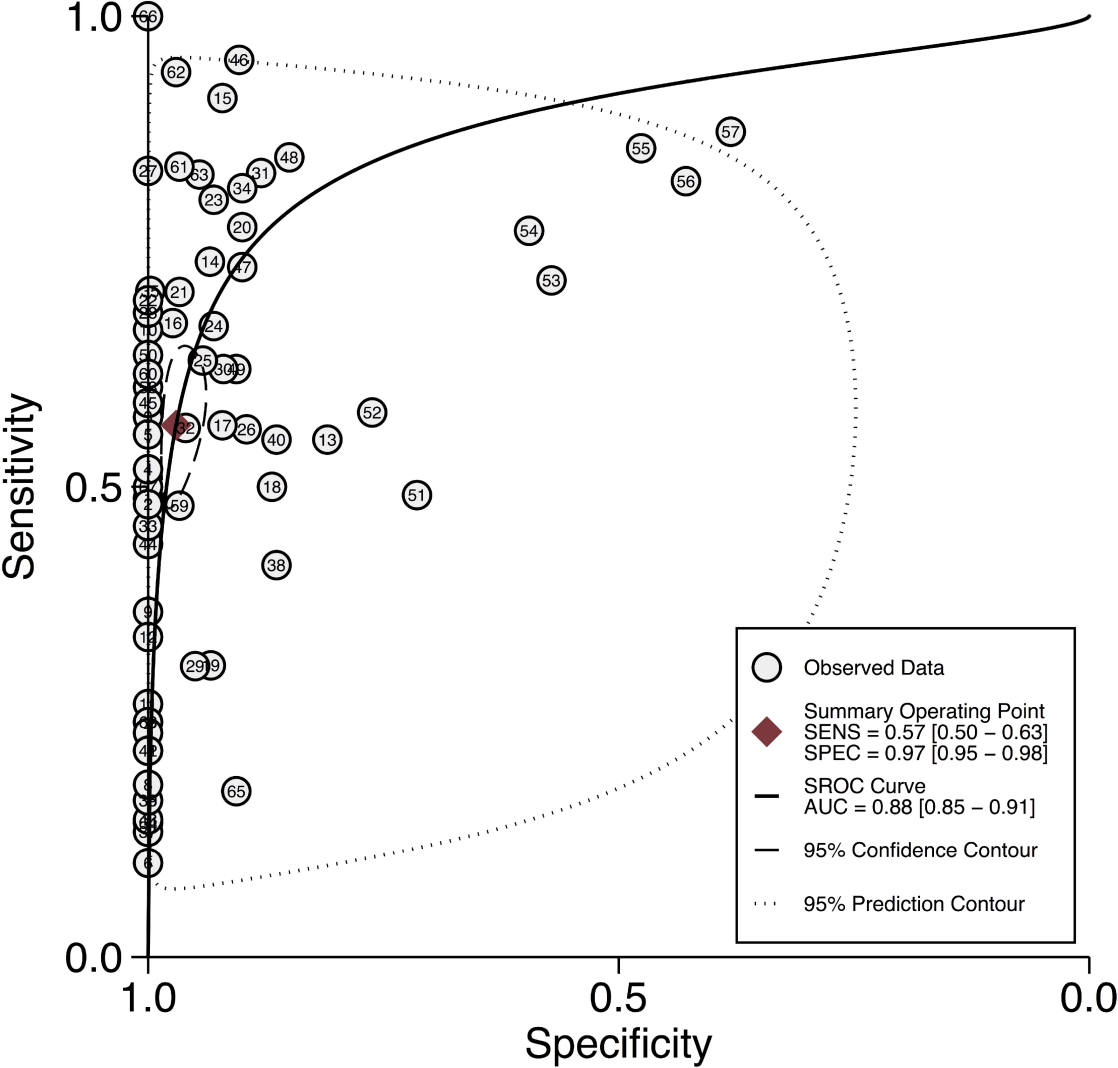
Summary ROC curve with confidence intervals and prediction regions around mean operating sensitivity and specificity points for detection of gastric cancer

Forest plot of individual and pooled sensitivities and specificities of all DNA methylation markers for diagnosing patients with gastric cancer are shown in Figure 2. The pooled sensitivity and specificity for detecting gastric cancer by the presence of one or more DNA methylation genes were 57% (95% CI 50–63%) and 97% (95% CI 95–98%), respectively. In addition, the pooled +LR (positive likelihood ratio) and -LR (negative likelihood ratio) were 19.1 (95% CI 11.0–33.0) and 0.45 (95% CI 0.38–0.52), respectively. The DOR (diagnostic odds ratio) was 42 (95% CI 24–74). The I^2^ values for sensitivity and specificity were 93.1% and 90.3%, respectively, implying significant heterogeneity between studies. Thus, the random effects model was used to pool the values. In addition, the SROC (summary receiver operating characteristic) curve for the included studies is presented in Figure 3. The AUC (area under the curve) was 0.88 (95%CI 0.85–0.91).

### Subgroup analysis

In order to explore the heterogeneity between studies, study endpoints were calculated using different subgroups listed in Table 2. First, we evaluated the diagnostic accuracy of DNA methylation by sample type. The pooled sensitivity of DNA methylation detection using serum was 50% (95% CI 43–58%), specificity was 98% (95% CI 96–99%), +LR was 25.0 (95% CI 13.2–47.5), -LR was 0.51 (95% CI 0.44–0.59), DOR was 49 (95% CI 25–95), and the AUC was 0.91 (95% CI 0.88–0.93). The pooled sensitivity of DNA methylation detection using plasma was 71% (95% CI 59–81%), specificity was 89% (95% CI 78–94%), +LR was 6.3 (95% CI 3.2–12.2), -LR was 0.33 (95% CI 0.23–0.47), DOR was 19 (95% CI 8–44), and the AUC was 0.87 (95% CI 0.84–0.89). The sensitivity of plasma-based testing was higher (71% vs. 50%; mean difference = 21%, 95% CI 17–23%) compared to serum-based testing. When assessing diagnostic accuracy by single compared to multiple genes methylation, the pooled sensitivity of single DNA methylation marker for detecting cancer was 52% (95% CI 45–60%), specificity was 98% (95% CI 96–99%), +LR was 21.4 (95% CI 12.0–38.3), -LR was 0.49 (95% CI 0.42–0.57), DOR was 44 (95% CI 24–81), and the AUC was 0.90 (95% CI 0.87 - 0.92). The pooled sensitivity of multiple DNA methylation markers for detecting gastric cancer was 76% (95% CI 64–84%), specificity was 85% (95% CI 65–95%), +LR was 5.2 (95% CI 2.1–12.8), -LR was 0.28 (95% CI 0.20–0.40), DOR was 18 (95% CI 7–47), and the AUC was 0.85 (95% CI 0.82–0.88). Presence of multiple DNA methylation showed higher sensitivity (76% vs. 52%; mean difference = 24%, 95% CI 21–27%) for detecting gastric cancer compared to the presence of single DNA methylation. Furthermore, we provided a subgroup analysis based on geographic regions: Asia (China, Japan, Thailand, Iran, South Korea, India, Singapore) vs. other Regions (Chile, Greece). The pooled sensitivity of DNA methylation in studies originating from Asia was 55% (95% CI 48–62%), specificity was 97% (95% CI 94–98%), +LR was 17.8 (95% CI 10.2–31.1), -LR was 0.47 (95% CI 0.40–0.54), and DOR was 38 (95% CI 22–67). The AUC was 0.87 (95% CI 0.84–0.89). The pooled sensitivity of DNA methylation in the studies originating from other regions was 80% (95% CI 61–91%), specificity was 99% (95% CI 66–100%), +LR was 120.1 (95% CI 1.7–8,579.0), -LR was 0.21 (95% CI 0.10–0.43), DOR was 586 (95% CI 10–35,903), and AUC was 0.97 (95% CI 0.95 - 0.98). The pooled sensitivity of DNA methylation marker was higher in the studies originating from other regions (80% vs. 55%; mean difference = 25%, 95% CI 95%CI 21–29%) compared to those from Asia.

**Table 2:** Pooled sensitivities and specificities of evaluation of DNA methylation by subgroups

	Sensitivity [95% CI]	Specificity [95% CI]	+LR [95% CI]	-LR [95% CI]	DOR [95% CI]	AUC [95% CI]
Overall	57% [50–63%]	97% [95–98%]	19.1[11.0–33.0]	0.45 [0.38–0.52]	42 [24−74]	0.88 [0.85–0.91]
Sample types						
Serum–based	50% [43–58%]	98% [96–99%]	25.0 [13.2–47.5]	0.51 [0.44–0.59]	49 [25−95]	0.91 [0.88–0.93]
Plasma–based	71% [59–81%]	89% [78–94%]	6.3 [3.2–12.2]	0.33 [0.23–0.47]	19 [8−44]	0.87 [0.84–0.89]
Number of markers						
Single	52% [45–60%]	98% [96–99%]	21.4 [12.0–38.3]	0.49 [0.42–0.57]	44 [24−81]	0.90 [0.87–0.92]
Multiple	76% [64–84%]	85% [65–95%]	5.2 [2.1–12.8]	0.28 [0.20–0.40]	18 [7−47]	0.85 [0.82–0.88]
Stage						
Early (I + II)	55% [47–64%]	96% [92–98%]	12.9 [6.6–25.0]	0.47 [0.39–0.56]	28 [13−59]	0.85 [0.81–0.88]
Advanced (III + IV)	68% [60–75%]	96% [92–98%]	15.5 [7.9–30.2]	0.33 [0.26–0.43]	46 [21−104]	0.91 [0.88–0.93]
Geographic regions						
Asia	55% [48–62%]	97% [94–98%]	17.8 [10.2–31.1]	0.47 [0.40–0.54]	38 [22−67]	0.87 [0.84–0.89]
Other regions	80% [61–91%]	99% [66–100%]	120.1 [1.7–8579.0]	0.21 [0.10–0.43]	586 [10−35903]	0.97 [0.95–0.98]

**Figure 4:**
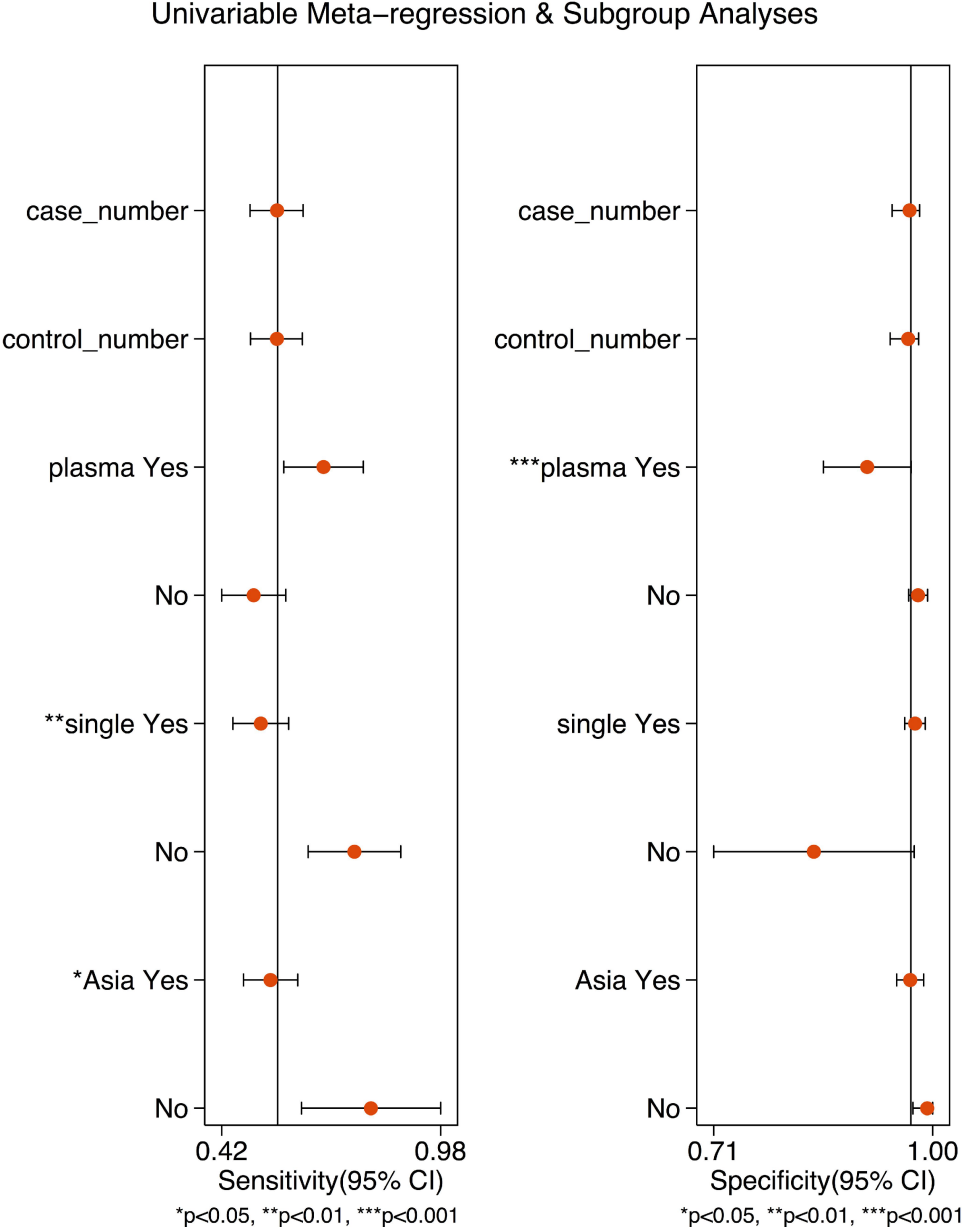
Forest plots of multivariable meta-regression and subgroup analysis for sensitivity and specificity

A meta-regression was conducted to assess potential sources of heterogeneity between studies by using following parameters (Figure 4): numbers of patients with gastric cancer and non-gastric cancer, DNA methylation markers profiling (single vs. multiple), test sample type (serum vs. plasma), and geographic regions (Asia vs. other regions). DNA methylation markers profiling (*P* < 0.01) and region of the study origination (*P* < 0.05) had an effect on sensitivity, and test sample type (*P* < 0.001) had an effect on specificity.

### Diagnostic accuracy by stage of gastric cancer

When early stage gastric cancer (TNM stage I +II) was evaluated as an outcome, the pooled sensitivity of DNA methylation for detecting early gastric cancer was 55% (95% CI 47–64%), specificity was 96% (95% CI 92–98%), +LR was 12.9 (95% CI 6.6–25.0), -LR was 0.47 (95% CI 0.39–0.56), DOR was 28 (95%CI 13–59), and the AUC was 0.85 (95% CI 0.81–0.88). When advanced stage gastric cancer (TNM stage III + IV) was evaluated as an outcome, the pooled sensitivity of DNA methylation detecting advanced stage gastric cancer was 68% (95% CI 60–75%), specificity was 96% (95% CI 92–98%), +LR was 15.5 (95% CI 7.9–30.2), -LR was 0.33 (95% CI 0.26–0.43), DOR was 46 (95% CI 21–104), and the AUC was 0.91 (95% CI 0.88–0.93). The pooled sensitivity of DNA methylation marker for detecting early gastric cancer was lower (55% vs. 68%; mean difference = 13%, 95% CI 11–15%) compared to detecting advanced gastric cancer.

### Assessment of bias

**Figure 5:**
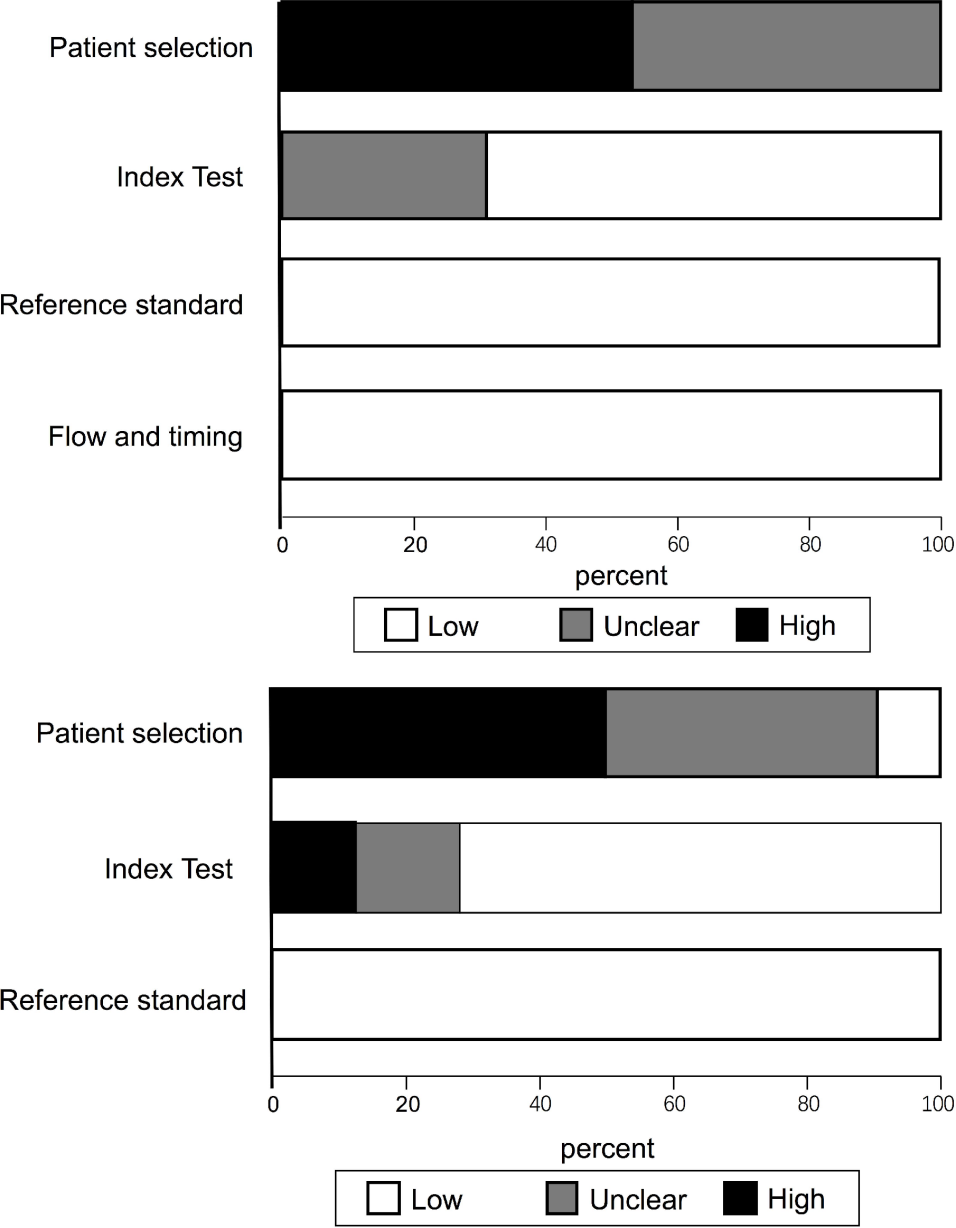
Overall quality assessment of included studies (QUADAS-2 tool)

**Figure 6:**
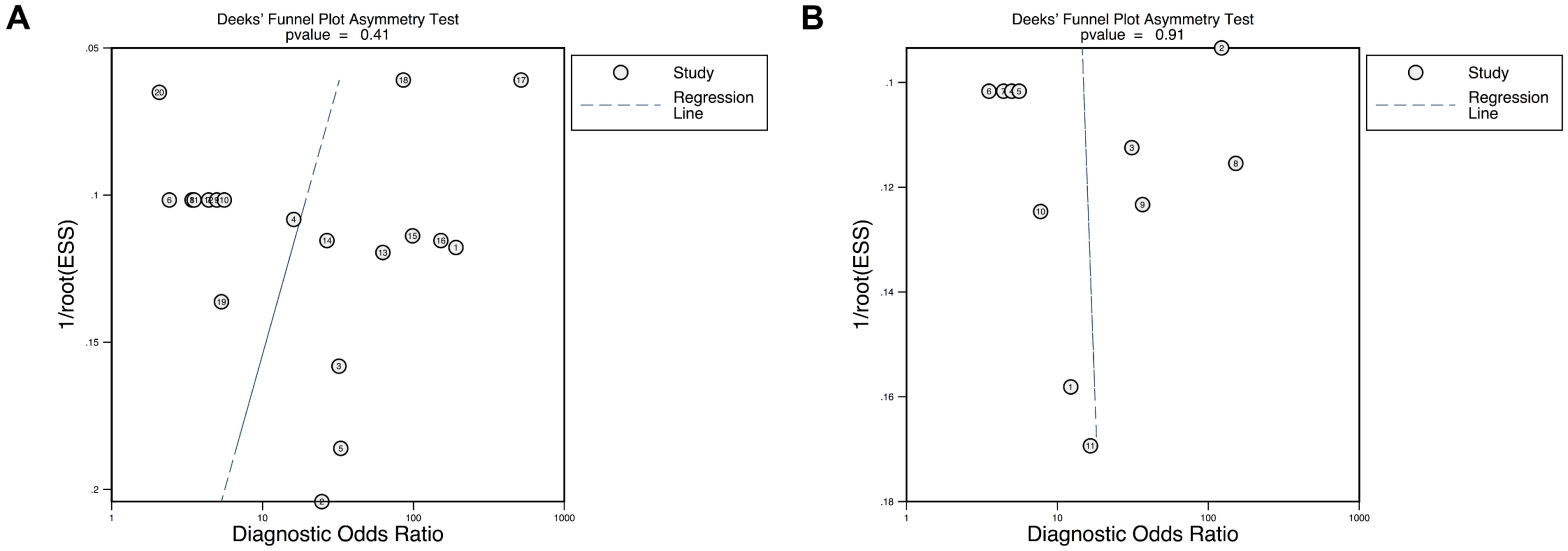
Deeks’ funnel plot asymmetry test to assess publication bias in estimates of diagnostic odds ratio for (**A**) plasma-based testing, (**B**) presence of multiple DNA methylation markers.

The quality assessment showed presence of high or unclear risk of bias for patient selection given that all the studies were case-control study design (Figure 5). 10 studies did not clarify whether the index test was interpreted prior to the knowledge of the reference standard. All studies showed low risk of bias in the categories of reference standard and flow & timing domains. In addition, publication bias was detected by Deeks’ funnel plot asymmetry test (*P* < 0.05). However, the subgroup analyses of plasma testing (*P* = 0.41) and multiple DNA methylation markers (*P* = 0.91) did not demonstrate publication bias (Figure 6).

## DISCUSSION

DNA methylation refers to the addition of a methyl-group to the carbon 5’ position of the cytosine ring of CpG dinucleotides to form 5-methylcytosine. CpG dinucleotides are concentrated in the upstream promoter region of many genes [15]. Aberrant methylation of the promoter region involved in the inactivation of tumor suppressor gene plays an important role in the tumorigenesis of multiple cancers (head and neck cancer, colon cancer, bladder cancer) and appears to be promising biomarkers for cancer detection [16–19]. Emerging studies have also shown that tumor suppressor genes (e.g. RNF180, Zic1) are frequently methylated, not only in gastric cancer, but also among patients with pre-malignant gastric lesions. Therefore, dysregulation in CpG-island methylation is likely to be involved in the early stages of gastric carcinogenesis, and DNA methylation may be utilized to detect early stage gastric cancer.

In the present meta-analysis, we evaluated the diagnostic accuracy of methylated genes for detecting gastric cancer from plasma or serum specimens. Overall, DNA methylation detection in the peripheral blood of gastric cancer patients exhibited a potential diagnostic utility given a modest sensitivity of 57% (95% CI 50–63%), high specificity of 97% (95% CI 95–98%), and a moderate-to high AUC of 0.88 (95% CI 0.85–0.91), which are superior to other conventional cancer markers. For example, a meta-analysis reported the pooled sensitivities of 21% for CEA, 28% for CA19-9, and 30% for CA72-4 for detecting gastric cancer. Although these blood biomarkers are commonly used in clinical practice, sensitivities for detecting early stage cancer are even lower, ranging from 9–23% [20]. Serum pepsinogen is another widely used biomarker to diagnose gastric cancer. A recent meta-analysis evaluating the combination of serum pepsinogen I levels and serum pepsinogen I/II ratio for detecting gastric cancer showed a sensitivity of 70% (95% CI 66–75%), specificity of 79% (95% CI 79–80%), and the AUC of 0.78 (95% CI 0.72–0.81) [21].

Furthermore, we performed additional analysis to determine whether the diagnostic utility of DNA methylation markers differed among certain subgroups. We found that sensitivity of DNA methylation was higher among plasma-based (71%) compared to serum-based testing (50%) with similar specificities. In addition, multiple DNA methylation markers had higher sensitivity (76%) compared to a single DNA methylation marker (52%). Therefore, a panel of DNA methylation genes may improve current limitations of marginal sensitivity in detecting gastric cancer for clinical use.

Finally, we assessed diagnostic accuracy of DNA methylation markers in detecting early stage of gastric cancer. The pooled sensitivity of DNA methylation detection to detect early stage gastric cancer (TNM stage I + II) was 55%, specificity was 96% and the AUC was 0.85, respectively, while the pooled sensitivity of DNA methylation detection among advanced gastric cancer (TNM stage III + IV) was 68%, specificity was 96% and the AUC was 0.91, respectively. Our results suggest that the DNA methylation markers evaluated to date are limited in diagnosing early stage gastric cancer which is important for an optimal screening test.

Blood-based testing for detecting gastric cancer is advantageous over conventional strategy of screening endoscopy given greater convenience, superior safety, and lower cost when applying to a large screening population [22]. However, currently available biomarkers (CEA, CA 19-9, serum pepsinogen I/II ratio) lack sufficient sensitivity and specificity for detecting early gastric cancer. Recently, microRNA as an emerging biomarker for detecting gastric cancer reported a pooled sensitivity of 78% (95% CI 73–81%), specificity of 80% (95% CI 76–84%) and the AUC of 0.86 (95% CI 0.83–0.89) respectively [23]. Although the sensitivity of microRNA appears superior to DNA methylation markers, chemically instability of RNA compared to DNA may pose technical challenges as a diagnostic test. Given current limitations, a combination of DNA methylation test with other blood-based biomarkers may enhance the sensitivity for detecting gastric cancer and increase its utility as a screening test.

The majority of the studies represented in our meta-analysis utilized methylation-specific PCR (MSP) to determine the methylation status of blood specimens. Although MSP is low-cost, which is an important factor for an optimal screening test, evaluation of only one or two CpG sites is possible which may impact the sensitivity. Although bisulfite sequencing that evaluates methylation status of each CpG locus has superior precision and is considered the gold standard, the application in clinical practice is currently limited by complexity and high-cost. However, improvement in the evaluation of DNA methylation technique in the future may increase generalizability as a screening test for gastric cancer [24, 25].

Our meta-analysis has limitations. First, all the studies evaluating DNA methylation status as a diagnostic test for gastric cancer included in the meta-analysis were case-control studies, rather than cohort studies, which have a higher risk of bias. Second, substantial publication bias was detected by Deeks’ funnel plot asymmetry test in the primary analysis. However, the subgroup analyses of plasma-based testing and evaluating multiple DNA methylation markers did not demonstrate evidence of publication bias supporting validity. Subgroup analyses results further suggested that the mode of testing, stage of patients, DNA methylation markers profiles, and the region of study may explain for the study heterogeneity, which was additionally confirmed by our meta-regression analysis. Third, a small number of patients from regions other than Asia and those with methylation in the promoter of multiple genes included in the meta-analysis may affect the generalizability of our findings. Our results should be interpreted with caution and will require validation in future population based-studies that encompass patients screening for gastric cancer.

In conclusion, blood based DNA methylation test had high specificity but modest sensitivity for detecting gastric cancer. Utilizing a combination of multiple compared to single gene methylation tests and evaluating plasma compared to serum sample may improve the diagnostic sensitivity.

## MATERIALS AND METHODS

A comprehensive literature search using Pubmed, Cochrane, Web of science, and Embase for relevant articles to November 20, 2016 was conducted to identify studies assessing diagnostic accuracy of DNA methylation utilizing plasma or serum in patients with gastric cancer. The following search strategies were used: (((((“Stomach Neoplasms” [Mesh]) OR (“gastric neoplasm*” OR “gastric cancer” OR “gastric tumor” OR “gastric carcinoma” OR “gastric oncology*” OR “stomach neoplasm*” OR “stomach cancer” OR “stomach tumor” OR “stomach carcinoma” OR “stomach oncology*”))) AND ((“DNA Methylation”[Mesh]) OR (methylation*))) AND ((“Diagnosis”[Mesh]) OR (test* OR discover* OR find* OR detect* OR diagnosis* OR screen* OR biomarker OR marker))) AND (blood OR plasma OR serum OR sera). The references of relevant publications were also thoroughly searched for additional studies.

### Study criteria

We included articles that met the following criteria: 1) patients with gastric cancer, 2) assays evaluating DNA methylation markers in blood specimens, 3) sensitivity and specificity values for detection of gastric cancer reported or calculable from the primary data. We excluded articles with any of the following criteria: 1) meeting abstracts, reviews, letters, comments, editorials, and meta-analysis; 2) evaluation of outcomes other than gastric cancer; 3) evaluating prognosis of patients with established gastric cancer; 4) non-English manuscript.

### Data extraction

Data including name of the lead author, publication year, country of study origination, types of samples examined, analyzed genes, experimental methods, sample size, and frequency of DNA methylated status in cases and controls were extracted from the selected study.

### Quality assessment

All publications that met our inclusion criteria were evaluated by QUADAS-2 guidelines. [26] Two authors (ZWF, LQF) independently abstracted and assessed the risk of bias for each study using standardized methods that include four key domains: patient selection, index test, reference standard, flow and timing (Supplementary Table 1).

### Study measures

The primary endpoint was sensitivity and specificity of blood DNA methylation tests for detecting gastric cancer. Secondary endpoints included +LR, -LR, DOR of blood DNA methylation tests. In addition, primary and secondary endpoints were calculated in subgroups by blood specimen types (serum vs. plasma), geographic region of study origination (Asian vs. other regions), number of DNA methylation (single vs. multiple), and cancer stage (early vs. advanced stage). Early stage gastric cancer was defined by TNM stage I or II while advanced stage cancer was defined by TNM III or IV. Finally, study endpoints were calculated using the stage of gastric cancer as outcomes (early vs. advanced stage).

### Statistical analysis

STATA 12.0 was used to perform the meta-analysis. Sensitivity, specificity, +LR, -LR, and DOR with corresponding 95% CI were calculated using a random effects model due to significant heterogeneity. A SROC curve was plotted based on each analysis, and the AUC was used to evaluate the overall diagnostic test accuracy. Furthermore, the I^2^ value was used to evaluate heterogeneity between studies. In addition, meta-regression and subgroup analysis were performed to identify the potential sources of heterogeneity. Presence of publication bias was evaluated by the Deeks’ funnel plot analysis.

## SUPPLEMENTARY MATERIALS TABLE


