# Supplementary material for: Diagnostic accuracy of DNA methylation in detection of gastric cancer: a meta-analysis
Source: Oncotarget. 2017 Nov 3;8(68):113142–52. doi: 10.18632/oncotarget.22613 (PMC5762578; doi:10.18632/oncotarget.22613)
Supplement: Supplementary file 1 [file oncotarget-08-113142-s001.pdf]

Diagnostic accuracy of DNA methylation in detection of gastric cancer: a meta-analysis

SUPPLEMENTARY MATERIALS

Supplementary Table 1: QUADAS-2: Risk of bias and applicability judgments

DOMAIN	PATIENT SELECTION	INDEX TEST	REFERENCE STANDARD	FLOW AND TIMING
Description	Describe methods of patient selection: Describe included patients (prior testing, presentation, intended use of index test and setting):	Describe the index test and how it was conducted and interpreted:	Describe the reference standard and how it was conducted and interpreted:	Describe any patients who did not receive the index test(s) and/or reference standard or who were excluded from the 2x2 table (refer to flow diagram): Describe the time interval and any interventions between index test(s) and reference standard:
Signalling questions(yes/no/unclear)	Was a consecutive or random sample of patients enrolled?	Were the index test results interpreted without knowledge of the results of the reference standard?	Is the reference standard likely to correctly classify the target condition?	Was there an appropriate interval between index test(s) and reference standard?
	Was a case-control design avoided?<br>Did the study avoid inappropriate exclusions?	If a threshold was used, was it pre-specified?	Were the reference standard results interpreted without knowledge of the results of the index test?	Did all patients receive a reference standard?<br>Did all patients receive the same reference standard?<br>Were all patients included in the analysis?
Risk of bias: High/low/unclear	Could the selection of patients have introduced bias?	Could the conduct or interpretation of the index test have introduced bias?	Could the reference standard, its conduct, or its interpretation have introduced bias?	Could the patient flow have introduced bias?
Concerns regarding applicability: High/low/unclear	Are there concerns that the included patients do not match the review question?	Are there concerns that the index test, its conduct, or interpretation differ from the review question?	Are there concerns that the target condition as defined by the reference standard does not match the review question?	
